# Biogenic and Synthetic Peptides with Oppositely Charged Amino Acids as Binding Sites for Mineralization

**DOI:** 10.3390/ma10020119

**Published:** 2017-01-28

**Authors:** Marie-Louise Lemloh, Klara Altintoprak, Christina Wege, Ingrid M. Weiss, Dirk Rothenstein

**Affiliations:** 1 Institute of Biomaterials and Biomolecular Systems (IBBS), Biobased Materials, University of Stuttgart, Pfaffenwaldring 57, 70569 Stuttgart, Germany; ingrid.weiss@bio.uni-stuttgart.de; 2 Institute of Biomaterials and Biomolecular Systems (IBBS), Molecular Biology and Plant Virology, University of Stuttgart, Pfaffenwaldring 57, 70569 Stuttgart, Germany; klara.altintoprak@bio.uni-stuttgart.de (K.A.); christina.wege@bio.uni-stuttgart.de (C.W.); 3 Projekthaus NanoBioMater, Allmandring 5B, 70569 Stuttgart, Germany; 4 Institute for Materials Science, Chair of Chemical Materials Synthesis, University of Stuttgart, Heisenbergstraße 3, 70569 Stuttgart, Germany; dirk.rothenstein@imw.uni-stuttgart.de

**Keywords:** inorganic-binding peptides, biomineralization, silicification, phage display

## Abstract

Proteins regulate diverse biological processes by the specific interaction with, e.g., nucleic acids, proteins and inorganic molecules. The generation of inorganic hybrid materials, such as shell formation in mollusks, is a protein-controlled mineralization process. Moreover, inorganic-binding peptides are attractive for the bioinspired mineralization of non-natural inorganic functional materials for technical applications. However, it is still challenging to identify mineral-binding peptide motifs from biological systems as well as for technical systems. Here, three complementary approaches were combined to analyze protein motifs consisting of alternating positively and negatively charged amino acids: (i) the screening of natural biomineralization proteins; (ii) the selection of inorganic-binding peptides derived from phage display; and (iii) the mineralization of tobacco mosaic virus (TMV)-based templates. A respective peptide motif displayed on the TMV surface had a major impact on the SiO_2_ mineralization. In addition, similar motifs were found in zinc oxide- and zirconia-binding peptides indicating a general binding feature. The comparative analysis presented here raises new questions regarding whether or not there is a common design principle based on acidic and basic amino acids for peptides interacting with minerals.

## 1. Introduction

Peptides which interact with inorganic materials are attractive tools for materials design and various fields of application. To name only a few, such peptides can serve as linkers applied for the immobilization of enzymes for the biochemical synthesis of (bioactive) compounds and in detection systems for sensing and medical diagnostics [1,2], or for the functionalization of biotemplates allowing selective and site-specific deposition of inorganic materials [3]. Further applications are surface engineering of protein–inorganic hybrid particles for biomedical and technical applications [4], and the generation of hybrid materials with peculiar properties introduced by biomineralization approaches [5,6].

Peptides bear an excellent degree of accuracy for positioning distinct functionalities at sub-nanometer level for nanoscale interaction with materials interfaces. Among those functionalities are hydrophilicity and hydrophobicity, and even features related to aromatic or ionic bonds can be very simply implemented based on the most common set of 20 natural amino acids. However, it is difficult to precisely determine or predict the interaction of peptides with the materials interface. One reason is that the functionality of the common amino acids is about an order of magnitude above that of distances of ions in a crystal. The largest amino acid, tryptophan, for example measures 1.2 nm in its longest dimension. All other biogenic amino acids are smaller. The ion radii of ammonia and calcium are 143 pm and 106 pm, respectively, and those of carbonate, sulfate, hydroxide and chloride are 178 pm, 232 pm, 133 pm, and 181 pm, respectively. Consequently, there is a spatial discrepancy so that not each functional group of consecutive amino acid residues in a protein may contribute to the binding of the inorganic phase and a straightforward matching of the functional groups of the organic and inorganic phase is not possible.

In contrast to crystals, however, peptides are polymers with some degree of flexibility. Protein flexibility is limited because peptide bonds are stabilized due to partial sp2 hybridization. Conversely, some functional groups are more flexible due to the presence of the aliphatic side chain (“spacer”) between the functional side group and the peptide backbone. For example, the so-called “acidic” amino acids aspartic acid and glutamic acid differ by just one –CH_2_ unit. The “spacers” of so-called “basic” amino acids lysine and arginine are much larger, but in contrast to lysine, which contains a single terminal amino group, arginine carries a more complex guanidine group with three nitrogen atoms in total. However, both of them carry on average one positive net charge at physiological pH values.

The different material and peptide features raise several questions, how the presence of either one or several of the amino groups and their possible degrees of freedom, and the peptide itself influence or even determine their binding to materials at either already existing interfaces or during the process of materials formation. In view of the fact that many natural composite materials are known to contain organic molecules that carry charged functional groups, the present paper focuses on peptides that contain charged amino acids.

The special focus is on material-binding peptides which contain both, positively and negatively charged amino acids. Here, we describe three different strategies to identify such peptides. Each strategy is based on its own experimental approach. The analysis presented here is based on the presence of oppositely charged amino acids within a relatively short part of a protein or peptide sequence. The comparative sequence analysis of two completely unrelated proteins from a natural calcium carbonate (CaCO_3_) biomineralization process, which will be briefly outlined in the next section as an example, raised three main questions that guided us through this paper:

1. Do we find oppositely charged amino acids in other natural biomineralization proteins, and is this a characteristic feature of species- and/or mineral-specific biomineralization processes, especially with respect to CaCO_3_?
2. Is there a general mode of action of peptides carrying oppositely charged amino acids to interact with materials other than the natural SiO_2_ or CaCO_3_ biominerals? In particular, how frequently do peptides identified by means of phage display and material screening approaches exhibit such properties?
3. Is it possible to transfer features such as oppositely charged residues known from other natural mineral systems (exemplified for domains contributing to SiO_2_ formation here), to guide and control mineral deposition onto heterologous carrier templates such as tobacco mosaic virus (TMV)-based nanotubes and rings, thereby reducing the degrees of freedom of the peptides immobilized on TMV?

Besides providing a common basis for tackling a sophisticated problem from different sides, our second aim is to point out or even create new synergistic effects between these strategies. These strategies can be further used to identify new interesting peptide motifs and may also be exploited for predicting and design inorganic-binding peptides and their properties with respect to functional material interactions.

## 2. Materials and Methods

### 2.1. Sreening of Biomineralization Proteins

**Figure 1:**
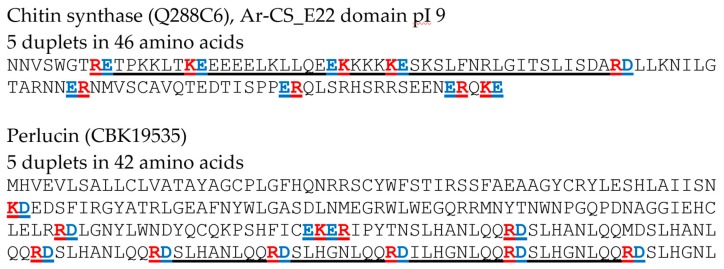
Examples for oppositely charged amino acid motifs in biomineralization proteins. R/K-E/D duplets are indicated (red and blue). Sequence region containing five such duplets are underlined (46 amino acids for chitin synthase domain and 42 amino acids for perlucin), see also Table 1.

First, protein sequences of chitin synthase Ar-CS1 (*Atrina rigida*) (tr|Q288C6) E22 pI 9 domain and *Haliotis laevigata* (CBK19535) perlucin, were analyzed with respect to the distribution of R/K-E/D duplets (R (Arg), K (Lys), E (Glu), and D (Asp)) and the lengths of a sequence region containing 5 duplets of R/K-E/D was calculated (number of amino acids) (Figure 1). Based on the findings, we defined the screening criteria for other biomineralization proteins as: minimum of 5 duplets of R/K-E/D within 50 amino acids (minimum of 20% charged amino acids). Second, the screening for sequence motifs containing duplets of R/K-E/D within biomineralization protein sequences was performed based on the database BioMine-DB (www.biomine.net). The file “biominproteins” was analyzed with respect to proteins that fulfill the criterion of a minimum of 5 duplets of R/K-E/D within 50 amino acids. Selected examples of such proteins are listed in Table 1 and the analyzed sequences are provided as Supplementary Material (S1). Proteins that fulfill this criterion were further analyzed regarding: (a) the exact length of the sequence motif containing 5 duplets (number of amino acids); (b) the pI; (c) the number of R, K, D, E; and (d) mol% of charged amino acids (R, K, E, D, B (Asx), H (His), and Z (Glx)) of both, the motif and the whole sequence using EMBOSS Pepstats.

### 2.2. Phage Display

Briefly, the inorganic-binding peptides were selected by phage display from a commercially available pseudo-random 12-mer peptide library (New England Biolabs, Inc.). As inorganic target substrates single crystals of (0001) ZnO, (000-1) ZnO, and (100) yttria-stabilized (9.5 mol%) zirconia (YSZ) (CrysTec, Berlin, Germany) were applied. The substrates were equilibrated in TBS (Tris buffered saline), pH 7.5, 0.1% Tween 20 for one hour at RT. Then, the peptide library (1.5 × 10^11^ phages) was bound to the target substrates for one hour at RT. Afterwards, the substrates were extensively washed to eliminate unbound phage/peptides. Interacting peptides were eluted either chemically or physically. A minimum of three biopanning rounds were performed to select the inorganic binding peptides. Selected phage clones were amplified and the DNA was extracted using standard methods for sequencing.

### 2.3. Re-Evaluation of Inorganic-Binding Peptides

Based on the findings of the duplet motif in natural biomineralization proteins, the ZnO- and ZrO_2_-binding peptides, selected by phage display, were re-evaluated on the presence of the duplet motif. The isoelectric point (pI) and the charge of the peptide at a given pH were calculated with Vector NTI software (Invitrogen) and Protein Calculator (http://protcalc.sourceforge.net/).

### 2.4. Silicification on TMV-Coupled Peptides

Details are provided as Supplementary Material S2.

## 3. Results

### 3.1. Oppositely Charged Amino Acid Motifs in Biomineralization Proteins

Possible strategies to identify mineral and metal binding motifs in natural systems include screenings of biomineralization related proteins and their binding domains. During the process of biomineralization, proteins play important roles in regulating mineralization and material properties. These proteins interact with the organic and mineral matrices and their precursors. Especially their mineral binding motifs are of great interest. However, common binding principles and motifs are hard to identify since biomineralization proteins interact with a range of different materials, at different environmental conditions and at various levels of regulation [7,8]. They can function as enzymes or structural proteins [9], promote or inhibit crystal growth and they can be incorporated as intracrystalline biomolecules [10]. Available data on biomineralization proteins offer the possibility to compare mineral-associated proteins and to search for unifying principles like conserved sequence regions, certain domains like C-type lectin domains, acidic or disordered domains, to mention only a few. For metazoan CaCO_3_ skeletons many of these aspects were recently discussed by Marin and co-authors [11].

Two thoroughly studied examples of proteins involved in biomineralization from mollusks are chitin synthase as a protein involved in regulation and formation of chitin and the second example, perlucin as a matrix protein. Chitin synthase Ar-CS1 (*Atrina rigida*) was studied in more detail [12,13,14]. As a biomineralization transmembrane protein, the different domains might interact with the organic (chitin) and mineral (CaCO_3_) phase as well as with other factors relevant for regulation, like pH, ion concentration, or mechanical properties of the mineral. For chitin synthase Ar-CS1 especially the extracellular E22 pI 9 domain displayed relatively high numbers of acidic and basic amino acids as well as R/K-E/D duplets (R (Arg), K (Lys), E (Glu), and D (Asp)) (Figure 1) [13]. An accumulation of acidic and basic amino acids and duplets was also observed for perlucin from *Haliotis laevigata* that incorporates into CaCO_3_ in vivo and in vitro (Figure 1) [15,16,17].

Based on these observations we searched for amino acid sequence regions which contain R/K-E/D duplets. The file “biominproteins” of a database for biomineralization proteins (BioMine-DB, biomine.net) was screened for such motifs. This data file was created based on extensive literature analysis on proteins involved in the biomineralization process [18]. Based on the sequences for chitin synthase Ar-CS1_E22 pI 9 domain and perlucin, we screened for proteins that fulfill the criterion of a minimum of 5 duplets of R/K-E/D within 50 amino acids (minimum of 20% charged amino acids) (see Figure 1). Examples for biomineralization proteins that were identified based on these criteria are given in Table 1.

Interestingly, regions with high numbers of acidic and basic amino acids as well as R/K-E/D duplets can be found in quite different biomineralization proteins. Examples listed in Table 1 include regulatory proteins (e.g., calcium-sensing and transport) as well as biomineral matrix proteins (e.g., osteopontin and N16.5). The length of the sequence regions containing 5 duplets varied between 26 and 50 amino acids and the isoelectric point (pI) for these regions was between 3.77 and 11.16 (Table 1). The pI of the full sequence and fragments analyzed varied in between 4.12 and 10.22 and these proteins contain about 20–40 mol% charged amino acids (Table 1). This first analysis of charged amino acids and duplets in biomineralization proteins revealed that such motifs might be involved in the regulation and interaction with biominerals. However, a more in depth analysis of these sequence regions is required and protein tertiary structure, known functional protein domains and the pH conditions have to be taken into account.

### 3.2. Inorganic Binding Peptides Identified by Phage Display

**Figure 2:**
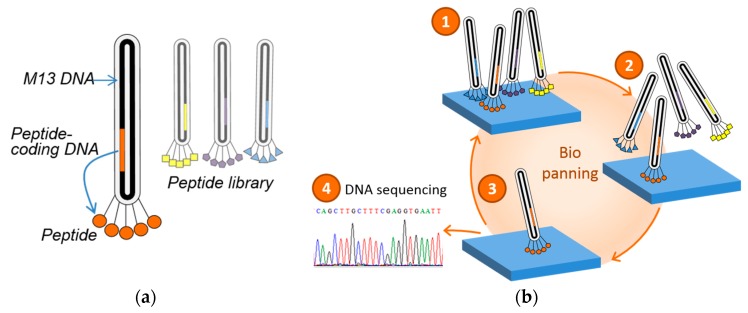
Scheme of phage display technique: (**a**) the peptide library is expressed fused to the minor coat protein pIII; and (**b**) selection process for the identification of interacting peptides, so called biopanning. (1) Incubation of the peptide library with target substrate; (2) non-interacting phages are eliminated from the peptide library; and (3) strongly bound phages are isolated. To increase the binding specificity, Steps 1 to 3 are repeated. Identification of the peptide sequence by sequencing the corresponding DNA fragment.

The screening of natural mineralization proteins revealed the short duplet motif consisting of oppositely charged amino acids. In general, such short motifs are difficult to identify by sequence alignments in biomineralization proteins as well as in inorganic binding peptides selected on binding to inorganic materials. However, knowing the duplet motif, a pool of inorganic binding peptides can be screened for its presence and thus conclude on the universality of this motif and expand the validity of the binding motif to non-natural materials. In recent years, the adaption of biomineralization processes for the formation of technical materials has come in the focus of material science. In general, a specific biomolecule modulates, control, or template the mineralization of the inorganic materials. As effector molecules like in the natural system, peptides were often selected. For the identification of peptides that specifically interact with technical inorganic materials, biochemical selection methods such as the phage display technology was applied. In phage display, peptides are selected from a pseudo-random peptide library based on binding to the inorganic substrate (Figure 2). Briefly, the peptide library is expressed on the surface of the phage as fusion to a coat protein. For the successful surface expression in M13 phages the corresponding DNA sequences of the peptides were inserted into the genes of the coat proteins p3, p8, p7, and p9, resulting in a phage clone expressing one specific peptide. The final peptide library comprises several 10^9^ various phage clones presenting each a defined peptide.

Recent findings on natural biomineralization proteins draw the attention on duplets of oppositely charged amino acids [17]. Based on these findings previously identified ZrO_2_-binding peptides [5] and ZnO-binding peptides [19] were analyzed with regard to such amino acid duplets and a minimum of 20% (corresponding to 25% in a 12-mer peptide) of charged amino acid residues in the peptide sequences. These peptides were selected by phage display from single crystalline ZrO_2_ and ZnO substrates, respectively and were further applied for biomineralization approaches [4,5]. The base for the following analysis is the “technical diversity” of peptides [20], that means peptide sequences with multiple records were considered only once.

**Figure 3:**
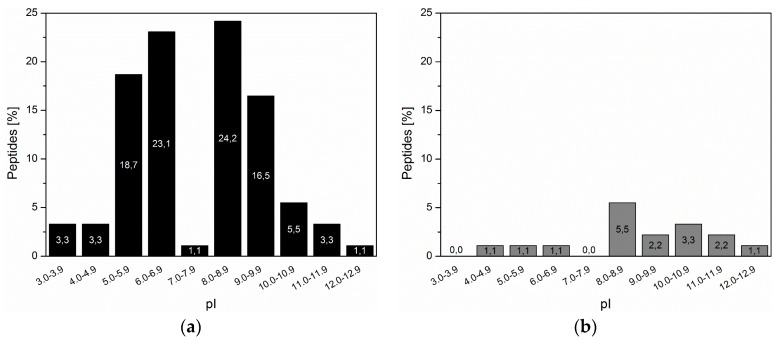
Percentage distribution of isolated ZrO_2_-binding peptides based on the calculated isoelectric point (pI): (**a**) percentage distribution of all selected peptides; and (**b**) percentage distribution of peptides with a duplet motif of the total number of peptides.

From the selected ZrO_2_-binding peptides, 17.6% (16 sequences) of the sequences were composed of at least 25% of charged amino acids (Table 2). From these sequences, seven out of 16 (43.75%) contained a duplet motif similar to natural biomineralization proteins. Basic (K and R) and acidic (D and E) amino acid residues did not show preferred combination and no preferred order, whether a basic or an acidic amino acid residues was on the first position. The isoelectric point (pI) of the highly-charged peptides was more often in the basic pH range starting from 8.0 and higher, e.g., 14.3% of high charged peptides had a pI ≥ 8.0 while of a mere 3.3% were below 7.0 (Figure 3). A similar distribution of pI was also reflected for the peptides with duplets, six out of nine peptides had a pI above 8.5.

The ZnO-binding peptides were less divers compared to the ZrO_2_-binding peptides. Four out of 29 peptides (13.8%) were highly charged with more than 20% charged amino acid residues (Table 3). Duplet arrangements were found in three of these four peptides. All of the duplets in the ZnO-binding peptides started with an acidic amino acid residue. Similar to the ZrO_2_-binding peptides, the majority of the pI of the highly charged ZnO-binding peptides was in the basic pH range. Mineralization of ZnO from zinc nitrate precursor solutions showed a strong influence of the duplet containing peptide ERSWTLDSALSM on the resulting crystallite size of ZnO [21]. Due to the interaction of the peptide with ZnO or intermediate products smaller crystallites were precipitated compared to reaction solutions without an inorganic-binding peptide.

In addition, for other inorganic-binding peptides interacting with Pd [22], Pt [22,23], Ag [24] a duplet motif in the peptide sequence is present. A seven-mer inorganic-binding peptide (LGFREKE) which was identified as strongest interacting peptide for amorphous and crystalline nickel boride (Ni_3_B) contained two duplet motifs in its sequence [25].

The relative level of each individual amino acid residue in the peptide library is firstly determined by the number of corresponding codons. The inorganic-binding peptides for both substrates are selected from a peptide library encoded by 32 codons and results in a biased amino acid residue abundance [20]. Unbalanced charge conditions in the peptide sequence, which result in a positive net-charge of the protein of +2 and higher, severely affects phage propagation in bacteria [26]. In addition, high numbers of only positively charged residues (≥2 arginine residues) markedly reduced the phage titer [26]. Concluding, that phage clones expressing peptides with a nearly balanced number of charged amino acid residues are supposed to amplify in bacteria normally levels. However, negative residues may not completely compensate the limitations opposed from positively charged amino acid residues. The peptides containing a duplet motif follow these rules of unbiased phage amplification, the net charge was between −1 and +1 and the peptide sequences contained low numbers of arginine residues. However, other peptide sequences with high numbers of arginine residues were nonetheless isolated.

The discovery of duplet containing biomineralization proteins in nature clearly inspires the bioinspired mineralization of technical materials concerning the screening of artificial mineralization peptides with regard to this novel sequence feature.

### 3.3. TMV-Coupled Bio-Inspired Peptides for Silicification

Plant viruses are widely used bio-templates for the nanometrically precise display of functional molecules, for purposes ranging from high-sensitivity biodetection devices up to cell culture supports for tissue engineering [27]. Aiming at intimate connections between biological and inorganic technical or implant materials, spatially selective mineral deposition has been investigated for differently shaped plant viruses including the filamentous *Potato virus X* [28], the spherical *Cowpea mosaic virus* [29,30,31,32] and the nanotubular *Tobacco mosaic virus* (TMV, [33,34,35,36,37]). TMV has emerged as an especially suitable scaffold in the field of bio-inspired mineralization, due to the dimensions and homogeneity of the viral particles, nanometrically precise arrangements of chemical groups, sustainable multiplication protocols, and an amenability to hierarchical combination with other material classes. Furthermore, both size and shape of TMV-derived particles are modifiable to a remarkable extent [38,39,40,41,42,43]. Recently, stable ring-like TMV-deduced structures have become accessible, which open up numerous possible uses as adapter modules in hybrid materials of complex functionality and for investigating site-selective mineral deposition [44]. With 18 nm outer diameter, a central 4 nm pore, and 10 nm length, they also might constitute attractive templates for a mass production of rigid nanorings, which are not available through simple batch fabrication routines so far.

Protein domains and peptides with numerous basic and acidic amino acid residues have been identified to promote mineralization processes of certain silica-precipitating organisms such as diatoms [45]. Among those, silaffins constitute a major protein class, with lysine- (Lys-) and serine- (Ser-)-rich amino acid sequences and a high extent of posttranslational modifications [46,47]. A predominant motif occurring inside the silaffins’ protein chain is K-(A/S/Q)-X-K, which determines the mode of modification of Lys with positively charged aminopropyl units and polyamines [48]. Additional negative charges may be introduced through phosphorylation of the frequently occurring amino acid residue Ser [47]. This results in a zwitterionic character of such protein domains and peptides proteolytically released thereof, and is proposed to be responsible for a self-assembly into structure-predefining matrices. Furthermore, the alternating arrangement of opposite charges seems to account for silica precipitation in correspondence with polyamines [47,49]. As these naturally occurring peptides are suitable candidates for hybrid material formation several trials had been conducted to evaluate the mineral deposition efficiency of silaffin-derived peptides [50,51,52].

Other silica-forming organisms, such as sponges produce the protein silicatein, which has been suggested to induce silica formation by an enzymatically driven condensation of silicic acid [53,54,55]. The catalytic activity is assumed to be based on a so-called charge relay effect, as it has also been proposed to explain the silica deposition induced by zwitterionic peptides with alternating Asp and His or Lys residues ((HD)_n_ and (KD)_n_ [56]), and by beta-sheet-forming peptides with a catalytic triad-like peptide monomer VHVEVS (Val-His-Val-Glu-Val-Ser) [57]. As previously demonstrated, peptide-equipped TMV particles are beneficial templates for a spatially selective silica precipitation directed to the multivalent outer surface of these richly available, robust nucleoprotein nanotubes [58]. This was investigated using a set of different peptides, out of which a peptide species with duplets of alternating basic (Lys, K) and acidic (Asp, D) amino acid residues (KDKDKDKDKDKDKDKDKDKDC [(KD)_10_C]) induced a reliable mineralization of peptide-fashioned TMV particles from a tetraethoxysilane (TEOS) precursor in ethanolic solution in a sol-gel process. Time course experiments over reaction periods of 12 days confirmed a reproducible progression of the mineralization, resulting in bioinorganic hybrid “nanosticks” with a TMV core of 18 nm diameter and silica shells of up to about 5 nm thickness [58].

**Figure 4:**
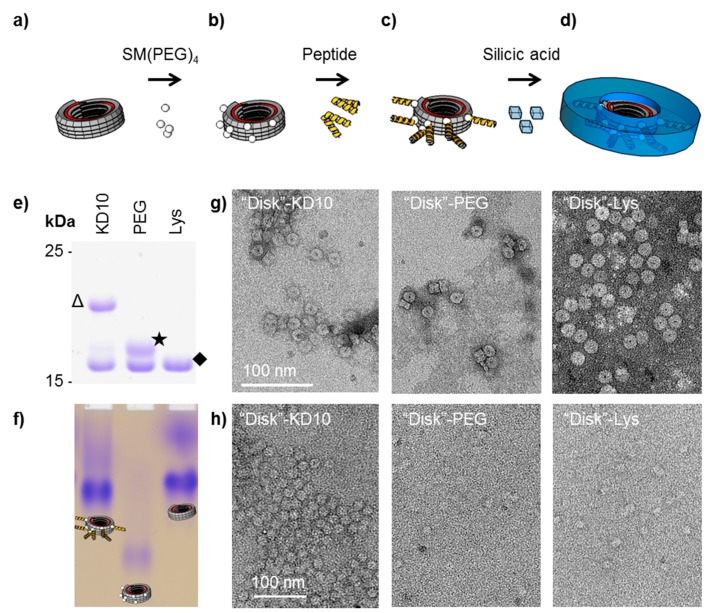
Mineralization of tobacco mosaic virus (TMV)-based nucleoprotein structures. Workflow and ring-shaped mineralization products obtained through peptide coupling and thus surface activation of TMV “disks”, and peptide-governed silica deposition. (**a**) Assembly of amino group-exposing engineered TMV CPs and a 204 nts RNA containing the TMV origin of assembly (OAs) into a short ring-like four-turn helix (“disk”-Lys). One amino group per CP is exposed to the outer ring surface allowing chemical modification. (**b**) Functionalization with hetero-bifunctional crosslinker molecules (succinimidyl-[(N-maleimidopropionamido)-tetratethyleneglycol] ester (SM(PEG)_4_)) results in “disk”-PEG; and (**c**) subsequent coupling of silica-deposition inducing peptide (KD)_10_C (yielding “disk”-KD_10_). (**d**) Silica shell formation guided by the peptide-equipped TMV-“disk” surface in the presence of hydrolyzed tetramethyl-orthosilicate (TMOS), i.e., a silicic acid substrate. (**e**) SDS-PAGE analysis indicates the proportion of chemically modified TMV CP after functionalization with the crosslinker (asterisk), and after peptide conjugation (triangle) compared to unmodified CP (diamond). Around 50% of the CPs are equipped with (KD)_10_C. (**f**) Native gel electrophoresis of intact “disks” displays altered electrophoretic mobility due to differently charged surfaces induced by the modifications. (**g**) TEM analysis of negatively stained (2% uranyl acetate) particles: “disks” are stable when diluted in deionized water. (**h**) TEM analysis of unstained “disks” exposed to silicic acid for 30 min: electron-dense contrast indicates mineralization only of the peptide-equipped “disk”-KD_10_.

Hence, we have transferred the mineralization concept established for full-length TMV particles to the new ring derivatives, in order to obtain mineralized ring-like structures with predictable silica deposition. Whereas a genuine TMV particle typically consists of a 6395 nucleotides (nts) single-stranded RNA and about 2130 coat proteins (CPs) arranged in a helical tube-like structure with a length of 300 nm, 18 nm diameter and an inner channel of 4 nm [59], the ring-shaped construct is an assembly of a short RNA of 204 nts that is fully encapsidated between ~68 CPs only (Figure 4a; for construct design and characterization see the Supplementary Material S2 and [44]). The holey disk-like structures (also referred to as “disk” in the following) were equipped on their outer rim with multiple (KD)_10_C peptides by the same two-step procedure as TMV before [58]. For the modification of individual adjacent CPs with peptides, a minimal distance of 2.5 to 3.4 nm from peptide to peptide is assumed, taking into account the repetitive CP arrangement in the right-handed TMV helix [60]. The length of each peptide connected to the “disk” subunits via a chemical linker is about 6 nm compared with the disk diameter of 18 nm.

The “disks” were generated with a TMV CP variant exposing amino groups [61], and were further functionalized with heterobifunctional crosslinkers (succinimidyl-[(N-maleimidopropionamido)-tetraethyleneglycol] ester (SM(PEG)_4_)) and purified by gel filtration (Figure 4b). These maleimide-displaying “disks” (“disk”-PEG) were conjugated with (KD)_10_C peptides via the thiol groups of their terminal cysteines (Figure 4c; for detailed experimental procedure refer to Supplementary Material S2). The degree of chemical modification and the structural identity of the “disks” stored in deionized water for three days was confirmed by SDS-PAGE analysis and native gel electrophoresis (see Figure 4e,f) and by TEM (Figure 4g). Fifty to sixty percent of the CPs were equipped with crosslinker and peptide, resulting in an electrophoretic mobility shift. Subsequent silica deposition according to [58], however, could not be achieved with the method developed for full-length TMV as the RNA-containing disk-shaped TMV derivatives did not withstand incubation with TEOS in 40% (v/v) ethanol (Supplementary Material S2). Thus the “disk” mineralization was performed from an alternative precursor preparation: silicic acid in deionized water at pH 5.5, without the addition of ethanol. Silicic acid was generated from tetramethyl-orthosilicate (TMOS) as described for silaffin-induced silica precipitation [46].

Only peptide-equipped “disks” (“disk”-KD_10_) showed an electron-dense contrast in TEM analysis (Figure 4h), whereas for control “disks” without any modification (“disk”-Lys) or with maleimide-exposing linkers alone (“disk”-PEG) only pale contours on the carbon film were detectable.

These results demonstrate that the outer surface of the non-modified amino group-exposing TMV “disk” that also displays both positively and negatively charged patches did not promote any pronounced mineralization under these conditions from a silicic acid precursor. However, the “charge-relay” peptide (KD)_10_C was efficient. These results support the benefits of zwitterionic peptides with alternating basic and acidic residues for the activation of otherwise inert surfaces, to induce and govern a site-directed silica deposition. The products—rigid, readily dispersed hybrid nanorings with inorganic surface layers—represent a novel type of nanoscale construction element awaiting integration into highly-structured composite matrices and mesoscale machines.

## 4. Discussion and Conclusions

We report on the identification of a small protein motif, named duplet motif, composed of oppositely charged amino acid residues. The duplet motif not only exists in natural biomineralization proteins but also in peptides capable for binding technical materials, indicating that the motif is present across systems. In experimental mineralization approaches, the peptides containing the duplet motif influence the mineralization process, e.g., the morphology, of the mineralized inorganic material.

Three different strategies with the aim to compare and combine their potential for deciphering universal signatures of material binding and deposition-guiding peptides were reported. The different strategies crossed four different materials systems, including CaCO_3_ and silicate as natural materials as well as zirconium dioxide and zinc oxide as non-natural materials. Biomineralization protein sequences containing the duplet motif originated from very different species and various mineral systems, suggesting that the duplet motif has evolved in various biological systems. Moreover, the proteins were also suggested to function in diverse fields like signal transduction pathways or as biomineral matrix proteins. However, more functional assays will be needed in order to identify the exact roles of these protein domains. Interestingly, peptides originate form selection experiments (phage display) also showed oppositely charged amino acid stretches resembling the duplet motif. The fact that non-natural materials were used for their selection demonstrates that the presence of oppositely charged amino acids within short sequence parts may favor materials interaction in general, and that those examples found in natural systems may basically take advantage of a more general driving force. The functionality of duplet motifs was analyzed in silicification experiments. Synthetic peptides with repetitive duplets of oppositely charged amino acids, immobilized on TMV-based nanotemplates strongly influenced the in vitro mineralization process.

Since the duplet motif was only recently recognized, the exact mechanism of action has to be further investigated. In particular, the different degrees of freedom at the level of single amino acid residues are in the focus of research. Precise mechanisms, binding constants, as well as morphologies of mineral deposits are difficult to analyze. Possible approaches for biomineralization proteins/peptides include potentiometric titration assay [17], biosensors [14], and electron microscopy of crystallites [21]. Hypothetic models include attraction and repulsion, with spatial arranged charge carriers of the inorganic phase, considering the different length scales of charge distances of peptides and minerals. In addition, indirect effects, e.g., interaction with the surrounding media or precursor molecules, can be electrostatically enriched creating a pH-depend microenvironment favoring the mineralization process. Such an effect was shown on mixed NH^3+^/COOH^−^ terminated surfaces where SiO_2_ is preferentially nucleated and deposited [62]. Another study demonstrated an influence of zwitterionic chitosan backbone on silicification, presumably by forming hydrogen bonds and thus stabilizing silicate in solution [63,64]. A possible mechanistic explanation was presented for proteins with a so-called charge relay effect resulting from duplet motifs which were proposed to promote the dehydration of Si(OH)_4_ [57]. Our findings that duplet motifs of charged amino acids seem to interact with very different material systems will now enable further studies focusing on the mechanistic questions.

Combining database mining of natural systems, experimental screening on a peptide level, and functional characterization of tailored sequences in systems with different degrees of freedom and length scales enable the identification of hardly accessible protein motifs of the mineralization process, which might be otherwise overseen. This is a good example of the mutual stimulation of natural and technical systems towards the biotechnological exploitation and applications of naturally and synthetic material-binding peptides. This fruitful combination of strategies may lead to more universal binding motifs, starting from naturally inspired motifs as shown here for the example of oppositely charged amino acid pairs. The fact that similar binding motifs were identified in non-natural systems by large-scale phage display methodologies demonstrates that our novel approach for defining distinct, but non-common criteria for the total number/percentage and arrangement of charged amino acids within short sequence fragments is indeed straight-forward. Oppositely, these perhaps common principles could be useful not only for non-natural materials libraries, but as well for biomineral screenings. Sequence information obtained by such a step-wise and cross-interacting strategy will hopefully soon lead to fundamental knowledge at the functional level and, at the same time, provide a promising approach for application-driven research and mining of the recently very common, but by far under-explored biomineralization databases.

## Figures and Tables

**Table 1:** Examples of biomineralization proteins that contain acidic/basic amino acids as five R/K-E/D duplets in maximum 50 amino acids. aa^1^: number of amino acids of the region containing five R/K-E/D duplets (R (Arg), K (Lys), E (Glu), and D (Asp)), mol% charged: charged amino acids (R, K, E, D, B (Asx), H (His), and Z (Glx)) per selected sequence region/total sequence, aa^3^: total number of amino acids of the analyzed sequence. Sequences were selected from the BioMine-database that contains full sequences and fragments. *Atrina regida* (tr|Q288C6), *Haliotis laevigata* (CBK19535), *Bos taurus* (sp|P35384), *Rattus norvegicus* (sp|Q63803. sp|P08721. tr|F1LP22. tr|F1LRM7), *Danio rerio* (tr|Q5U7A7), *Pinctada fucata* (sp|Q4KTY1. sp|097048. tr|Q1MW92), *P. margaritifera* (tr|Q1KZ60) and *Tetrahymena thermophila* (tr|Q7YW43). pI was calculated using EMBOSS Pepstats.

Species	Protein	Motifs							Sequences		
		aa^1^	pI	K	R	D	E	mol% Charged	aa^3^	pI	mol% Charged
*A. rigida*	Chitin synthase, At-CS_E22, pI9	46	9.87	10	3	2	9	52.2	102	9.80	41.2
*H. laevigata*	Perlucin C protein precursor	42	7.88	0	5	5	0	33.3	240	7.02	25.4
*B. taurus*	Extracellular Ca-sensing receptor	40	8.47	4	3	2	4	32.5	1085	6.05	21.3
*R. norvegicus*	Guanine nucleotide-binding protein	26	10.60	5	5	2	5	65.4	1144	4.45	26
*R. norvegicus*	Osteopontin	50	4.57	5	2	8	6	50.0	317	4.12	27.2
*R. norvegicus*	Plasma membrane Ca-transporting ATPase	37	10.92	5	6	2	5	48.6	1181	5.86	25.7
*R. norvegicus*	Collagen alpha-1(II) chain	40	10.48	3	5	4	2	35.0	1419	8.44	19.1
*D. rerio*	Exostosin-2	42	7.54	2	6	2	6	40.5	719	7.31	24.9
*P. fucata*	Serine/threonine-protein kinase H1	46	11.16	7	7	3	3	45.7	415	10.22	31.6
*P. fucata*	N16.5 matrix protein	29	6.34	3	4	4	3	48.3	129	6.46	28.7
*P. fucata*	Shematrin-5	34	3.77	0	5	12	1	52.9	278	7.59	23.4
*P. margaritifera*	Calconectin	30	4.75	4	2	6	1	43.3	92	6.65	41.3
*T. thermophila*	Motor kinesin-like protein	32	4.69	8	1	5	6	62.5	424	9.30	28.1

**Table 2:** ZrO_2_-binding peptides selected form a peptide library by phage display. Peptide sequences with ≥25% charged amino acid residues were analyzed for the presence of a duplet motif. Peptides with a lower content of charged amino acids did not show a duplet motif. Negatively charged amino acids are highlighted blue, and positively charged amino acids are highlighted red. Peptides are sorted by the isoelectric point (pI).

Sequence	Negatively Charged AA	Positively Charged AA	Duplet	Net Charge pH 7	pI^1^
APSQPKDEVTAY	2	1	Yes	−1	4.37
NPTLHQERMQDW	2	1	Yes	−1	5.32
DLNYFTLSSKRE	2	2	Yes	0	6.07
ILSTQDLKAKSS	1	2	No	+1	8.59
HYPTAKFHAERL	1	2	Yes	+1	8.60
NLPKRDGWPLPW	1	2	Yes	+1	8.75
YSLRADSRWMPS	1	2	No	+1	8.75
FHEKQLSGGRFG	1	2	Yes	+1	8.76
AIMGPRTVDRLP	1	2	Yes	+1	9.60
IHSLQPEVNVRR	1	2	No	+1	9.61
QHVYHPKPKASR	0	3	No	+3	10.29
HLPQKKKPPHAI	0	3	No	+3	10.30
HLPQKKKPPHAM	0	3	No	+3	10.30
NKWPLAHSQKKR	0	4	No	+4	11.26
KKRRRPSMYVPI	0	5	No	+5	11.73
RKKTPASRRPMR	0	6	No	+6	12.48

^1^ The isoelectric point (pI) was calculated with Vector NTI software, Invitrogen.

**Table 3:** ZnO-binding peptides selected form a peptide library by phage display. Peptide sequences with ≥25% charged amino acid residues were analyzed for the presence of the duplet motif. Negatively charged amino acids are highlighted blue, and positively charged amino acids are highlighted red. Peptides are sorted by the isoelectric point (pI).

Sequence	Negatively Charged AA	Positively Charged AA	Duplet	Net Charge pH 7	pI ^1^
ERSWTLDSALSM	2	1	Yes	−1	4.37
MKPDKAIRLDLL	2	3	Yes	+1	8.59
HYPTAKFHAERL	1	2	Yes	+1	8.60
HHTHRVDVHQTR	1	2	No	+1	9.62

^1^ The isoelectric point (pI) was calculated with Vector NTI software, Invitrogen.

## Supplementary Materials

S1: Analyzed protein sequences presented in Table 1, S2: Detailed materials and methods for silicification experiments on TMV-derived ring-like nucleoprotein “disks”.

Supplementary File 1

Supplementary File 2
