# Supplementary material for: Biogenic and Synthetic Peptides with Oppositely Charged Amino Acids as Binding Sites for Mineralization
Source: Materials (Basel). 2017 Jan 28;10(2):119. doi: 10.3390/ma10020119 (PMC5459154; doi:10.3390/ma10020119)
Supplement: Supplementary File 1 [file materials-10-00119-s001.docx]

**Supplementary Materials: Biogenic and Synthetic Peptides with Oppositely Charged Amino Acids as Binding Sites for Mineralization**

Marie-Louise Lemloh, Klara Altintoprak, Christina Wege, Ingrid M. Weiss and Dirk Rothenstein

Supplementary Material S1:

Analyzed examples of protein sequences (from BioMine-database, file “biominproteins”) containing five duplets within 50 amino acids.

(1) Five duplets within 40 amino acids

**>sp|P35384|CASR_BOVIN Extracellular calcium-sensing Receptor OS=Bos taurus GN=CASR PE=2 SV=1**

MALYSCCWILLAFSTWCTSAYGPDQRAQKKGDIILGGLFPIHFGVAV**KD**QDLKSRPESVE

CIRYNFRGFRWLQAMIFAIEEINSSPALLPNMTLGYRIFDTCNTVSKALEATLSFVAQNK

IDSLNLDEFCNCSEHIPSTIAVVGATGSGISTAVANLLGLFYIPQVSYASSSRLLSNKNQ

FKSFLRTIPNDEHQATAMADIIEYFRWNWVGTIAADDDYGRPGI**EK**F**RE**EAE**ERD**ICIDF

SELISQYSDE**EK**IQQVVEVIQNSTAKVIVVFSSGPDLEPLI**KE**IVRRNITGRIWLASEAW

ASSSLIAMPEYFHVVGGTIGFGLKAGQIPGF**RE**FLQKVHPRKSVHNGFA**KE**FWEETFNCH

LQEGAKGPLPVDTFLRGHEEGGARLSNSPTAFRPLCTGEENISSVETPYMDYTHLRISYN

VYLAVYSIAHALQDIYTCIPGRGLFTNGSCADIKKVEAWQVLKHLRHLNFTSNMGEQVTF

DECGDLAGNYSIINWHLSPEDGSIVF**KE**VGYYNVYAKKG**ER**LFIND**EK**ILWSGFS**RE**VPF

SNCS**RD**CLAGTRKGIIEGEPTCCFECVECPDGEYSDETDASAC**DK**CPDDFWSNENHTSCI

A**KE**IEFLSWTEPFGIALTLFAVLGIFLTAFVLGVFIKFRNTPIVKATN**RE**LSYLLLFSLL

CCFSSSLFFIGEPQDWTCRLRQPAFGISFVLCISCILVKTNRVLLVFEAKIPTSFHRKWW

GLNLQFLLVFLCTFMQIVICAIWLNTAPPSSYRNHELEDEIIFITCHEGSLMALGFLIGY

TCLLAAICFFFAFKSRKLPENFNEAKFITFSMLIFFIVWISFIPAYASTYGKFVSAVEVI

AILAASFGLLACIFFNKVYIILFKPSRNTIEEVRCSTAAHAFKVAARATLRRSNVSRQRS

SSLGGSTGSTPSSSISSKSNSEDPFPQQQPKRQKQPQPLALSPHNAQQPQPRPPSTPQPQ

PQSQQPPRCKQKVIFGSGTVTFSLSFDEPQKTAVAHRNSTHQTSLEAQKNNDALTKHQAL

LPLQCGETDSELTSQETGLQGPVGEDHQLEMEDPEEMSPALVVSNSRSFVISGGGSTVTE

NMLRS

(2) Five duplets within 26 amino acids

**>sp|Q63803|GNAS1_RAT Guanine nucleotide-binding protein G(s) subunit alpha isoforms XLas OS=Rattus norvegicus GN=Gnas PE=1 SV=3**

MGMLNCLHGNNMSGQHDIPPEVGDQPEQEPLEAQGAAAPGAGVGPAEEMETEPSNNEPIP

DETDSEVCGPPEDSKSDIQSPSQAFEEVQVGGDYSPPPEEAMPFEIQQPSLGDFWPTLEQ

PGPSGTPSGIKAFNPAILEPGTPTGAHPGLGAYSPPPEEAMPFEFNEPAQE**DR**CQPPLQV

PDLAPGGPEAWVSRALPAEPGNLGFENTGF**RE**DYSPPPEESVPFQLDGEEFGGDSPPPGL

PRVTPQIGIGGEFPTVAVPSTLCLAPAANAPPLWVQGAIGRPF**RE**AVRSPNFAYDISPME

ITRPLLEIGRASTGVDDDTAVNMDSPPIASDGPPIEVSGAPVKSEHAKRPPL**ER**QAAETG

NSPISSTTAEEAKVPSL**ER**GEGSPTQPETVHIKPAPVAESGTDSSKADPDSATHAVLQIG

PEEVGGVPTMPTDLPPASEDAGPDVRAEPDGGTAPATPAESEDN**RE**PAAAAAAEPAAEPA

AEPAAEPAAEPAAEPAAEAVPDTEAESASGAVPDTQEEPAAAAASATPAEPAARAAPVTP

TEPATRAVPSARAHPAAGAVPGASAMSAAARAAAARAAYAGPLVWGARSLSATPAARASL

PARAAAAARAASAARAVAAGRSASAAPSRAHLRPPSPEIQVADPPTPRPAPRPSAWP**DK**Y

**ER**GRSCCRYEAASGICEIESSSDESEEGATGCFQWLLRRNRRPGQPRSHTVGSNPVRNFF

ARAFGSCFGLSECTRSRSLSPGKA**KD**PME**ER**RKQMR**KE**AMEM**RE**QKRA**DK**KRSKLI**DK**QL

EE**EK**MDYMCTHRLLLLGAGESGKSTIVKQMRILHVNGFNGEGGEEDPQAARSNSDG**EK**AT

KVQDIKNNL**KE**AIETIVAAMSNLVPPVELANPENQFRVDYILSVMNVPNFDFPPEFYEHA

KALWEDEGVRACY**ER**SNEYQLIDCAQYFL**DK**IDVIKQADYVPSDQDLPRCRVLTSGIFET

KFQV**DK**VNFHMFDVGGQ**RDER**RKWIQCFNDVTAIIFVVASSSYNMVI**RE**DNQTNRLQEAL

NLFKSIWNNRWLRTISVILFLNKQDLLA**EK**VLAGKSKIEDYFPEFARYTTPEDATPEPGE

DPRVTRAKYFI**RD**EFLRISTASGDGRHYCYPHFTCAVDTENIRRVFNDC**RD**IIQRMHLRQ

YELL

(3) Five duplets within 50 amino acids

**>sp|P08721|OSTP_RAT Osteopontin OS=Rattus norvegicus GN=Spp1 PE=1 SV=2**

MRLAVVCFCLFGLASCLPVKVAEFGSSE**EK**AHYSKHSDAVATWLKPDPSQKQNLLAPQNS

VSSEETDDFKQETLPSNSNESHDHMDDDDDDDDDGDHAESEDSVNSDESDESHHSDESDE

SFTASTQADVLTPIAPTVDVPDGRGDSLAYGLRSKSRSFPVSDEQYPDATDEDLTSRMKS

QESDEAIKVIPVAQRLSVPSDQDSNGKTSHESSQLDEPSVETHSLEQS**KE**YKQRASHEST

EQSDAIDSA**EK**PDAIDSA**ER**SDAIDSQASSKASLEHQSHEFHSHE**DK**LVLDPKS**KE**D**DR**Y

LKFRISHELESSSSEVN

(4) Five duplets within 37 amino acids

**>tr|F1LP22|F1LP22_RAT Plasma membrane calcium-transporting ATPase 2 OS=Rattus norvegicus GN=Atp2b2 PE=4 SV=2**

MGDMTNSDFYSKNQRNESSHGGEFGCSMEELRSLMELRGTEAVVKI**KE**TYGDTESICRRL

KTSPVEGLPGTAPDL**EK**RKQIFGQNFIPPKKPKTFLQLVWEALQDVTLIILEIAAIISLG

LSFYHPPGESNEGCATAQGGAEDEGEAEAGWIEGAAILLSVICVVLVTAFNDWS**KEK**QFR

GLQSRIEQEQKFTVVRAGQVVQIPVAEIVVGDIAQIKYGDLLPADGLFIQGNDLKIDESS

LTGESDQVRKSV**DKD**PMLLSGTHVMEGSGRMVVTAVGVNSQTGIIFTLLGAGGEEE**EKKD**

KKGVKKGDGLQLPAADGAAPANAAGSANASLVNGKMQDGSADSSQSKAKQQDGAAAMEMQ

PLKSAEGGDAD**DK**KKANMHK**KEK**SVLQGKLTKLAVQIGKAGLVMSAITVIILVLYFTVDT

FVVNKKPWLTECTPVYVQYFVKFFIIGVTVLVVAVPEGLPLAVTISLAYSVKV**RE**GKSRV

SPAQATHLSPQPP**EKE**GALPRQVGNKTECGLLGFVLDLRQDYEPVRSQMPE**EK**LYKVYTF

NSVRKSMSTVIKMPDESFRMYSKGASEIVLKKCCKILSGAGEPRVFRP**RDRD**EMVKKVIE

PMACDGLRTICVAY**RD**FPSSPEPDWDNENDILNELTCICVVGIEDPVRPEVPEAIRKCQR

AGITVRMVTGDNINTARAIAIKCGIIHPGEDFLCLEG**KE**FNRRIRN**EK**GEIEQ**ER**I**DK**IW

PKLRVLARSSPT**DK**HTLVKGIIDSTHTEQRQVVAVTGDGTNDGPALKKADVGFAMGIAGT

DVA**KE**ASDIILTDDNFSSIVKAVMWGRNVYDSISKFLQFQLTVNVVAVIVAFTGACITQD

SPLKAVQMLWVNLIMDTFASLALATEPPTETLLLRKPYGRNKPLISRTMMKNILGHAVYQ

LTLIFTLLFVG**EK**MFQIDSGRNAPLHSPPSEHYTIIFNTFVMMQLFNEINARKIHG**ER**NV

FDGIFRNPIFCTIVLGTFAIQIVIVQFGGKPFSCSPLQLDQWMWCIFIGLGELVWGQVIA

TIPTSRLKFL**KE**AGRLTQ**KE**EIPEEELNEDVEEIDHA**ERE**LRRGQILWFRGLNRIQTQIR

VVKAFRSSLYEGL**EK**PESRTSIHNFMAHPEFRIEDSQPHIPLIDDTDLEEDAALKQNSSP

PSSLNKNNSAIDSGINLTTDTSKSATSSSPGSPIHSLETSL

(5) Five duplets within 40 amino acids, Five duplets within 50 amino acids

**>tr|F1LRM7|F1LRM7_RAT Collagen alpha-1(II) chain OS=Rattus norvegicus GN=Col2a1 PE=4 SV=1**

MIRLGAPQSLVLLTLLIATVLQCQGQDARKLGPKGQKGEPGDI**KD**IIGPKGPPGPQGPAG

EQGPRG**DR**G**DK**G**ER**GAPGPRG**RD**GEPGTPGNPGPPGPPGPPGPPGLGGGNFAAQMAGGFD

**EK**AGGAQMGVMQGPMGPMGPRGPPGPAGAPGPQGFQGNPGEPGEPGVSGPMGPRGPPGPA

GKPGDDGEAGKPGKAG**ER**GLPGPQGARGFPGTPGLPGVKGHRGYPGLDGAKGEAGAPGVK

GESGSPGENGSPGPMGPRGLPG**ER**GRTGPAGAAGARGNDGQPGPAGPPGPVGPAGGPGFP

GAPGAKGEAGPTGARGPEGAQGSRGEPGNPGSPGPAGASGNPGTDGIPGAKGSAGAPGIA

GAPGFPGPRGPPGPQGATGPLGPKGQTGEPGIAGFKGEQGPKGETGPAGPQGAPGPAGEE

GKRGARGEPGGAGPIGPPG**ER**GAPGNRGFPGQDGLAGPKGAPG**ER**GPSGLAGPKGANGDP

GRPGEPGLPGARGLTGRPGDAGPQGKVGPSGAPGEDGRPGPPGPQGARGQPGVMGFPGPK

GANGEPGKAG**EK**GLAGAPGLRGLPG**KD**GETGAAGPPGPSGPAG**ER**GEQGAPGPSGFQGLP

GPPGPPGEGGKQGDQGIPGEAGAPGLVGPRG**ER**GFPG**ER**GSPGAQGLQGPRGLPGTPGTD

GPKGAAGPDGPPGAQGPPGLQGMPG**ER**GAAGIAGPKG**DR**GDVG**EK**GPEGAPG**KD**GGRGLT

GPIGPPGPAGANG**EK**GEVGPPGPSGSTGARGAPG**ER**GETGPPGPAGFAGPPGADGQPGAK

GDQGEAGQKGDAGAPGPQGPSGAPGPQGPTGVTGPKGARGAQGPPGATGFPGAAGRVGPP

GSNGNPGPAGPPGPAG**KD**GPKGARGDTGAPGRAGDPGLQGPAGAPG**EK**GEPGDDGPSGSD

GPPGPQGLAGQRGIVGLPGQRG**ER**GFPGLPGPSGEPGKQGAPGASG**DR**GPPGPVGPPGLT

GPAGEPG**RE**GSPGADGPPG**RD**GAAGVKG**DR**GETGALGAPGAPGPPGSPGPAGPTGKQG**DR**

GEAGAQGPMGPSGPAGARGIAGPQGPRG**DK**GEAGEPG**ER**GLKGHRGFTGLQGLPGPPGPS

GDQGTSGPAGPSGPRGPPGPVGPSG**KD**GSNGIPGPIGPPGPRGRSGETGPAGPPGNPGPP

GPPGPPGPGIDMSAFAGLGQ**REK**GPDPLQYMRADEADSTLRQHDVEVDATLKSLNNQIES

IRSPDGSRKNPARTCQDLKLCHPEWKSGDYWIDPNQGCTLDAMKVFCNMETGETCVYPNP

ATVPRKNWWSSKS**KEK**KHIWFGETMNGGFHFSYGDGNLAPNTANVQMTFLRLLSTEGSQN

ITYHCKNSIAYLDEAAGNLKKALLIQGSNDVEMRAEGNSRFTYTAL**KD**GCTKHTGKWGKT

IIEYRSQKTSRLPIVDIAPMDIGGPDQEFGVDIGPVCFL

(6) Five duplets within 42 amino acids

**>tr|Q5U7A7|Q5U7A7_DANRE Exostosin-2 OS=Danio rerio GN=ext2 PE=2 SV=1**

MCASGKYGSRGPALIPRMKTKHRIYYITLFSVVLLGLIATGMFQFWPHSIESSAEWSL**DR**

RSVHDAPLVRISVNSPIPGRGDLSCRMHTCFDVYRCGYNPKNKIKVYIYPLQRFVDEVGV

PISSTGLS**RE**YNDLLSAISDSDFYTDDVSRACLFVPSIDVLNQNSLRI**RE**TAQALAMLPR

W**DK**GMNHLLFNMLPGGPPDYNTALDVP**RDR**ALLAGGGFSTWTYRQGYDVSIPVYSPLSAE

VDLP**ER**QPGPRRYFILSSQTAIH**RE**YRVEL**ER**L**KD**ENGEALLLL**DK**CSNLSQGLTSVRKR

CYKGQVYDYPQILQESSFCVVLRGARLGQATLSDVLQAGCVPVIMADSYILPFSEVLDWK

RASVVIPE**EK**LPEMYTILKSIPHRQVEEMQRQARWFWEAYFSSMKAIGMTTLQIIN**DR**IY

PYAAHTYEEWNNPPVVKWSSVNSPLFLPLIPPRSPGFTAVVLTY**DR**IESLFRVITEISKV

PSLAKLLVVWNNQNKSPPEESLWPKVAVPLKVVRT**KE**NKLSNRFFPFDEIETEAVLAIDD

DIIMLTSDELQFGYEVW**RE**FP**DR**LVGYPGRLHLWDHEMGKWKYESEWTNEVSMVLTGAAF

YHKYFNYLYTYKMPGDIKNWVDAHMNCEDIAMNFLVANITGKAPIKVTPRKKFKCPECTA

IDGLSLDQTHMV**ER**SECINKFASVFGTMPLKVVEHRADPVLY**KD**DFP**EK**LKSFPNIGSL

(7) Five duplets within 46 amino acids

**>sp|Q4KTY1|KPSH1_PINFU Serine/threonine-protein kinase H1 homolog OS=Pinctada fucata GN=PSKH1 PE=2 SV=1**

MGCMSSKLVPEGPSGNQAVVEVFNN**ER**N**KE**YNRQNPHQNRGP**RD**PT**KD**PKNAGPPPEGQR

SNRKVKKY**RDK**FDPRVTAKYDIKALIGRGNFSKVVRVEHRVTKQPYAIKMI**DR**VQG**KE**VF

ESEVAVLRRVKHSYIIQLIEVFET**KDK**VYMVMELATGGELF**DR**IIAKGSFT**ERD**ATRVLN

MVLDGVKYLHGLGITH**RD**LKPENLLYYHPGHDSKIMITDFGLSSTRKGPENFMRTTCGTP

EYIAPEIIARKPYMCQVDMWAVGVITYILLSGTMPFDDENKTRLYRLILKAKYSYAGEHW

**KD**VSAQA**KD**FI**DK**LLVVSPG**DR**LSAADALKHQWLISNAASSSNKNLHRTISQNLIHRQST

RANSTKSAKSTRSTKSNKSNRSGRSLRSEHRRVMPDEIDELH**RD**PDVQADLASLG

(8) Five duplets within 29 amino acids

**>sp|O97048|MA165_PINFU N16.5 matrix protein OS=Pinctada fucata PE=1 SV=1**

MTCTLRWTITALVLLGICHLARPAFRTKCGRYSYCWIPYDI**ERDR**YDNG**DK**KCCFCRNAW

SPWQC**KE**D**ER**YEWLRCGHKFYYMCCYTDDDNGNGNGNGNGFNYLKSLYGGYGNGNGEFWE

EYID**ER**Y**DK**

(9) Five duplets within 34 amino acids

**>tr|Q1MW92|Q1MW92_PINFU Shematrin-5 OS=Pinctada fucata PE=2 SV=1**

MKFVTELVLLGLLCNICWCQIQRRRITWDGDCGDD**DRD**GYDDCSQNIGEDA**DRD**G**RD**DYT

GNCG**RD**VDGDG**RD**DCGGECADF**DRD**GSDDCFDDMDDAQGAYISPYLYRRRFGLGRFGLGR

FGLGRFGLGNPFMQNRQFGYGPIGGMNFRLGGLGYPYGRLGLGYGNLLSRYGGYGNILGG

YGNLRGLGGYGNLLGGYGSLRGYGNVGGYRGLGGYGNLYGGLGGYGNYGGYGHLGGYGYL

GGYRNLGGYGNYGLRPHGDNYGRYGVGSYLRRSRRKKY

(10) Five duplets within 30 amino acids

**>tr|Q1KZ60|Q1KZ60_PINMG Calconectin OS=Pinctada margaritifera PE=4 SV=1**

M**DK**IRSSPVSKK**RD**T**ER**AAPVGAPAD**DK**GKCQMSFS**DKD**N**DK**KLSEDEMTSILNDIQ**KDK**

KMFAKYDEDGNGFVDASEFSFKVAMEIRKCQK

(11) Five duplets within 32 amino acids

**>tr|Q7YW43|Q7YW43_TETTH C-terminal motor kinesin-like protein (Fragment) OS=Tetrahymena thermophila GN=Kin8 PE=3 SV=1**

FGFIFLQKRKKTFVLLFQIQIQFSNKQELRNKGETIEQLQNEI**KD**ITKK**KD**EEV**KE**L**KD**T

VDILTNKLDEET**KER**KILHNIVEDMKGKIRVFCRVRPPNENEVQMNSQNVVEVLDAMNCK

LQAKNGPKKFQFDSCSRQDDIFNDAKKLIQSAVDGYNVCIFAYGQTGSGKSFTMQGT**RE**M

PGITPRSVNELFNLLKPIQKTCKVTISAYIMELYMDNLIDLLAPPNSIMQKKLEI**KE**DYI

TNTTYVQNAT**KE**ELEQIIQKGILNRKISKTDMNVESSRSHLIITILINIFNPQTETTTHG

KISLIDLAGS**ER**ILKSGANPHQV**KE**ANSINKSLTALGDVISALTNQQQNGG**ER**HIPYRNN

KLTYLM**KD**SLGGNAKTLMIVNVSPSEYNLEETNSSLQYASRVKTIVNETSKNIET**KD**YTR

L**KEK**

Supplementary Material S2:

Effect of ethanolic tetraethoxysilane (TEOS) solution on TMV-derived “disks”

If TMV-based “disks” were subjected to an ethanol-containing tetraethoxysilane (TEOS) solution to serve as educt for mineralization, as performed in previous experiments with full-length TMV [[1](#_ENREF_1)], they completely disintegrated. This was confirmed by TEM analysis (Figure S1).


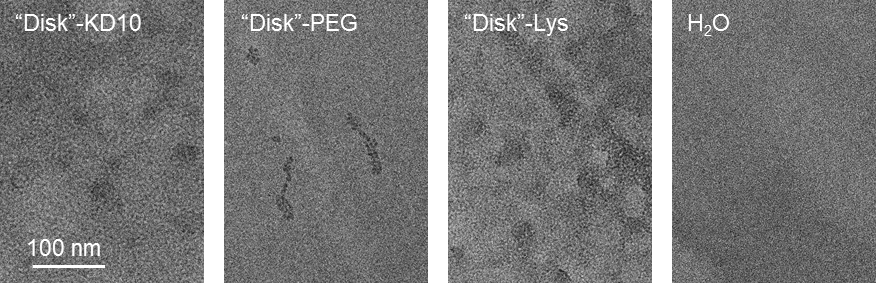


**Figure S1.** Remainder of TMV-derived nucleoprotein “disks” (as shown in Figure 4 g) after incubation in 10% TEOS in 40% ethanol for nine days at 25 °C, and corresponding control lacking “disks”. Supplementation of the TEOS solution with differently modified “disks” has led to unspecific silica precipitation detectable in all reactions (“Disk”-KD10: “disks” equipped with peptide (KD)_10_C via heterobifunctional crosslinker SM(PEG)_4_; “Disk”-PEG: “disks” functionalized with SM(PEG)_4_ only; and “Disk”-Lys: plain “disks” exposing amino groups accessible for chemical coupling). Different from “disks” incubated in water or silicic acid (Figure 4), no disk-like structures are visible anymore, indicating that they underwent denaturation under these conditions. The water control (H_2_O) without disks shows lower amounts of precipitates. TEM analysis of unstained products.

Materials and methods used for silica deposition on TMV-coupled peptides

Fabrication of RNA-stabilized TMV “disks”

TMV CP disks or proto-helices were stabilized with short RNAs of 204 nucleotides (nt) length containing the TMV OAs (referred to as 204^+^ nt RNA). The 204^+^ nt RNA was synthesized by in vitro transcription by means of a MEGAscript^®^ T7 High Yield Transcription Kit (Ambion, Austin, TX, USA). A short TMV cDNA fragment, containing the sequence of the TMV core Origin of Assembly (OAs position 5350-5531) [[2](#_ENREF_2)] fused to the T7 RNA polymerase promoter sequence [[3](#_ENREF_3),[4](#_ENREF_4)], of pGEM®-T Easy (Promega, Mannheim, Germany; construct I described in [[5](#_ENREF_5)]) was amplified by PCR and applied as in vitro transcription template (Table S1). In a 20 µL volume, 100 µg DNA-template was incubated for 6 h at 37 °C during the in vitro transcription reaction. The DNA-template was degraded by DNaseI treatment for 15 min at 37 °C. The synthesized RNA was precipitated by the addition of lithium chloride (final concentration (f. c.) 1.3 M), EDTA (f. c. 8.6 mM) and ethanol (f. c. 71% (*v*/*v*) over night at −20 °C. After centrifugation according to the supplier's information, the pellet was washed with 1 mL 70% (*v*/*v*) ethanol and dissolved in DMDC-(dimethyl dicarbonate)-treated deionized water (ddH_2_O; 18.3 MΩ cm; purified by a membraPure system, Aquintus, Bodenheim, Germany) to a final concentration of 3 µg/µL and stored at −80 °C. RNA-free TMV CP was prepared by acidic degradation of whole TMV particles [[6](#_ENREF_6)]. For “disk” assembly, a genetically engineered TMV mutant, TMV_Lys_, was used [[7](#_ENREF_7)]. A 10 mg/mL TMV_Lys_ solution was mixed in a 1:3 ratio (*v*/*v*) with glacial acetic acid and incubated for 20 min on ice. Released RNA was removed by centrifugation for 20 min at 20,000× *g* and 4 °C. The CP-containing supernatant was dialyzed against ddH_2_O in a dialysis tube (Spectra/Por^®^7 Dialysis Membrane, 8 kDa molecular weight cut-off [MWCO], Spectrum Laboratories, Rancho Dominquez, USA) with water changes every 8 h at 4 °C. As soon as the proteins started to flocculate (after 24 to 48 h), the dialysate was centrifuged as above. The resulting CP pellet was dissolved in 75 mM sodium potassium phosphate buffer (SPP) pH 7.2 and centrifuged for 10 min at 10,000× *g* to remove aggregated CP_Lys_. The supernatant was transferred to a new reaction tube. The CP concentration was determined by a NanoDrop ND-1000 spectrophotometer (PeqLab, Erlangen, Germany) at a wavelength of 280 nm, using the extinction coefficient of TMV CP [[1.3 mL·mg^−1^·cm^−1^; 8](#_ENREF_8)], adjusted to 10 mg/mL and incubated for at least 48 h at room temperature to allow disk formation according to Butler [[9](#_ENREF_9)]. For a typical assembly reaction, 66 µg RNA were incubated with 1000 µg CP_Lys_ with an f. c. of 0.9 µg/µL RNA and 6.8 mg/mL CP_Lys_ in 75 mM SPP (pH 7.2) for 16 h at 30 °C. The ring-shaped nucleoprotein assemblies of the 204^+^ nt RNA and about 68 CP subunits were stored in 75 mM SPP (pH 7.2) at 10 °C without any further purification and will be described in more detail elsewhere[[10](#_ENREF_10)].

**Table S1.** DNA templates for in vitro transcription of RNAs with (+) and without (−) the TMV origin of assembly (OAs). White marked letters indicate the major loop of the OAs, capital letters DNA sequence of viral genome OAs, small letters sequence of pGEM®-T Easy origin [[10](#_ENREF_10)].

| RNA Length [nt] | DNA-Sequence |
| --- | --- |
| 204^+^ | **gggcgaattgggcccgacgtcGCGGGTTTCTGTCCGCTTTCTCTGGAGTTTGTGTCGGTGTGTATTGTTTATAGAAATAATATAAAATTAGGTTTGAGAGAGAAGATTACAAACGTGAGAGACGGAGGGCCCATGGAACTTACAG*AAGAAGTTG*TTGATGAGTTCATGGAAGATGTCCCTATGTCGATCAGGCTTGCAAAGTTa** |

Functionalization of “disks” with mineralization-inducing peptides

RNA-stabilized “disks” were functionalized via the amino groups of the genetically modified CP_Lys_ subunits, which are exposed at the outer “disk” rim, with heterobifunctional crosslinker molecules. In a volume of 120 µL, RNA-stabilized “disks” (f. c. 5.1 mg/mL with regard to CP_Lys_ amount) in SPP (f. c. 75 mM, pH 7.2) were incubated with the bifunctional linker SM(PEG)_4_ (f. c. 1.2 mM, succinimidyl-[(N-maleimidopropionamido)-tetraethyleneglycol] ester; Thermo Scientific, Karlsruhe, Germany) stored in dimethyl sulfoxide (f. c. in the reaction 0.001%) at −20 °C) for 2 h under agitation (horizontal shaking at 500 rpm) at 30 °C. Excess crosslinker was removed by gel filtration using PD SpinTrap G-25 columns (GE Healthcare, Freiburg, Germany) which were equilibrated with 75 mM SPP at pH 7.2. This step was repeated twice. Subsequently, the purified crosslinker-functionalized “disks” (f. c. of 3.4 mg/mL), providing maleimide groups for chemical conjugation, were incubated in a total volume of 105 µL with 0.3 mg/mL (f. c.) (KD)_10_C (dissolved in dimethylformamide, f. c. in coupling reaction 0.05%) in SPP (f. c. 75 mM, pH 7.2) to couple the peptides via their thiol group at the C-terminal cysteine residue. The total volume of peptide-functionalized “disks” was purified by gel filtration as above. Immediately before mineralization, 75 mM SPP buffer (pH 7.2), containing functionalized or unmodified “disks” (in a volume of 105 µL with a f. c. of 2 mg/mL relating to the CP_Lys_ amount), was exchanged by gel filtration as above, however, using PD SpinTrap G-25 columns equilibrated with ddH_2_O.

Characterization of functionalized “disks”

The ratio of chemically modified to unmodified CP_Lys_ subunits per “disk” was determined by denaturing SDS-PAGE (sodium dodecyl sulphate polyacrylamide gel electrophoresis) according to Laemmli et al. [[11](#_ENREF_11)]. An amount of 3 µg “disks” was heated for 5 min at 95 °C in sample buffer (f. c.: 50 mM Tris-HCl (tris-(hydroxymethyl)-aminomethane hydrochloric acid) pH 6.8, 2% (w/v) SDS, 0.1% (w/v) bromophenol blue, 10% glycerol, 100 mM dithiothreitol) and separated on a 15% polyacrylamide gel. Proteins were fixed in the gels (10% acetic acid, 40% ethanol) for 15 min and stained with Coomassie Brilliant Blue R250 (Serva Electrophoresis, Heidelberg, Germany). The electrophoretic mobility of whole “disks” after chemical modification was compared to non-modified “disks” by native gel electrophoresis. Twelve-milligram “disks” were combined with sample buffer (f. c.: 10 mM SPP pH 7.2, 0.1% (w/v) bromophenol blue, 10% glycerol) and separated on a 2.7% agarose gel (Biozym Sieve 3:1 Agarose, Biozym, Hessisch Oldendorf, Germany) in 1 × TBE (89 mM Tris base, 89 mM boric acid, 2 mM EDTA). Proteins in the agarose gel were fixed and stained just as in the SDS-PAGE gels.

The structural integrity of the functionalized and unmodified “disks” stored in ddH_2_O for three days at 10 °C was determined by TEM analysis. A volume of 15 µL “disk” solution was dropped on Parafilm M^®^ (American National Can, Menasha, WI, USA) with a concentration of 0.05 mg/mL CP_Lys_. A carbon/Formvar^®^-covered 400-mesh copper grid (Science Service, Munich, Germany) was placed onto the droplet for 5 min. Excess solution was removed from the grids with filter paper, which were washed with three droplets of ddH_2_O. Samples on the grid were stained with 15 µL 2% (w/v) uranyl acetate for 3 min. After removing residual uranyl acetate with a filter paper, the grids were air-dried and analyzed with a Tecnai G2 Sphera electron microscope (FEI, Hillsboro, OR, USA) at 120 kV using a 16-megapixel camera TemCam F416 (TVIPS, Gauting, Germany).

Mineralization of functionalized “disks”

Chemically modified or plain “disks” were treated with TMOS using protocols established in this study on the basis of different references [[12–14](#_ENREF_12)]. For silicification with the starting compound TMOS, a silicic acid precursor solution was prepared (modified from [[12](#_ENREF_12)–[14](#_ENREF_14)]) by hydrolyzing 15 µL TMOS in 85 µL 1 mM HCl for 5 min at room temperature to obtain a 1 M silicic acid solution (final concentration of 0.85 mM HCl). The functionalized or unmodified “disks” (f. c. 1.3 mg/mL) in ddH_2_O were mixed with silicification solution to a f. c. of 20 mM hydrolyzed TMOS and 0.017 mM HCl with an ionic strength of 8.5 × 10^−9^ M and incubated for 30 min under agitation at 23 °C. Excess silicic acid was removed by dialysis against three times 500 mL ddH_2_O at 4 °C with ddH_2_O changes every 1 h, using Slide-A-Lyzer™ MINI Dialysis Devices (10K MWCO, Thermo Fischer Scientific, Darmstadt, Germany). Silica accumulation on the “disks” was analyzed by TEM as above, without uranyl acetate staining.

References

1. Altintoprak, K.; Seidenstucker, A.; Welle, A.; Eiben, S.; Atanasova, P.; Stitz, N.; Plettl, A.; Bill, J.; Gliemann, H.; Jeske, H.; et al. Peptide-equipped tobacco mosaic virus templates for selective and controllable biomineral deposition. *Beilstein J. Nanotechnol.* **2015**, *6*, 1399–1412.
2. Zimmern, D. An extended secondary structure model for the TMV assembly origin, and its correlation with protection studies and an assembly defective mutant. *EMBO J.* **1983**, *2*,
   1901–1907.
3. Rosa, M.D. Four T7 RNA polymerase promoters contain an identical 23 bp sequence. *Cell* **1979**, *16*, 815–825.
4. Panayotatos, N.; Wells, R.D. Recognition and initiation site for four late promoters of phage T7 is a 22-base pair DNA sequence. *Nature* **1979**, *280*, 35–39.
5. Schneider, A.; Eber, F.J.; Wenz, N.; Altintoprak, K.; Jeske, H.; Eiben, S.; Wege, C. Dynamic DNA-controlled “stop-and-go” assembly of well-defined protein domains on RNA-scaffolded TMV-like nanotubes. *Nanoscale* **2016**, *8*, 19853–19866.
6. Fraenkel-Conrat, H.; Williams, R.C. Reconstitution of active tobacco mosaic virus from its inactive protein and nucleic acid components. *Proc. Natl. Acad. Sci. USA* **1955**, *41*, 690–698.
7. Geiger, F.C.; Eber, F.J.; Eiben, S.; Müller, A.; Jeske, H.; Spatz, J.P.; Wege, C. TMV nanorods with programmed longitudinal domains of differently addressable coat proteins. *Nanoscale* **2013**, *5*, 3808–3816.
8. Raghavendra, K.; Adams, M.L.; Schuster, T.M. Tobacco mosaic virus protein aggregates in solution: Structural comparison of 20S aggregates with those near conditions for disk crystallization. *Biochemistry* **1985**, *24*, 3298–3304.
9. Butler, P.J. Structures and roles of the polymorphic forms of tobacco mosaic virus protein. VI. Assembly of the nucleoprotein rods of tobacco mosaic virus from the protein disks and RNA.
   *J. Mol. Biol.* **1972**, *72*, 25–35.
10. Altintoprak, K.; Seidenstücker, A.; Krolla-Sidenstein, P.; Plettl, A.; Jeske, H.; Gliemann, H.; Wege, C. RNA-stabilized bifunctional protein nanorings: High-precision adapter templates for bio-artificial hybrids. 2017, submitted.
11. Laemmli, U.K. Cleavage of structural proteins during the assembly of the head of bacteriophage T4. *Nature* **1970**, *227*, 680–685.
12. Kröger, N.; Deutzmann, R.; Sumper, M. Polycationic peptides from diatom biosilica that direct silica nanosphere formation. *Science* **1999**, *286*, 1129–1132.
13. Zane, A.C.; Michelet, C.; Roehrich, A.; Emani, P.S.; Drobny, G.P. Silica morphogenesis by lysine-leucine peptides with hydrophobic periodicity. *Langmuir: ACS J. Surf. Colloids* **2014**, *30*, 7152–7161.
14. Haase, N.R.; Shian, S.; Sandhage, K.H.; Kröger, N. Biocatalytic nanoscale coatings through biomimetic layer-by-layer mineralization. *Adv. Funct. Mater.* **2011**, *21*, 4243–4251.

© 2017 by the authors; licensee MDPI, Basel, Switzerland. This article is an open access article distributed under the terms and conditions of the Creative Commons by Attribution (CC-BY) license (http://creativecommons.org/licenses/by/4.0/).
